# The evolution of the tape measure protein: units, duplications and losses

**DOI:** 10.1186/1471-2105-12-S9-S10

**Published:** 2011-10-05

**Authors:** Mahdi Belcaid, Anne Bergeron, Guylaine Poisson

**Affiliations:** 1 Information and Computer Sciences, University of Hawaii, Honolulu, Hawaii, USA; 2 LaCIM, Université du Québec à Montréal, Canada

## Abstract

**Background:**

A large family of viruses that infect bacteria, called *phages*, is characterized by long tails used to inject DNA into their victims' cells. The *tape measure protein* got its name because the length of the corresponding gene is proportional to the length of the phage's tail: a fact shown by actually copying or splicing out parts of DNA in exemplar species. A natural question is whether there exist *units* for these tape measures, and if different tape measures have different units and lengths. Such units would allow us to retrace the evolution of tape measure proteins using their duplication/loss history. The vast number of sequenced phages genomes allows us to attack this problem with a comparative genomics approach.

**Results:**

Here we describe a subset of phages whose tape measure proteins contain variable numbers of an 11 amino acids sequence repeat, aligned with sequence similarity, structural properties, and simple arithmetics. This subset provides a unique opportunity for the combinatorial study of phage evolution, without the added uncertainties of multiple alignments, which are trivial in this case, or of protein functions, that are well established. We give a heuristic that reconstructs the duplication history of these sequences, using divergent strains to discriminate between mutations that occurred before and after speciation, or lineage divergence. The heuristic is based on an efficient algorithm that gives an exhaustive enumeration of all possible parsimonious reconstructions of the duplication/speciation history of a single nucleotide. Finally, we present a method that allows, when possible, to discriminate between duplication and loss events.

**Conclusions:**

Establishing the evolutionary history of viruses is difficult, in part due to extensive recombinations and gene transfers, and high mutation rates that often erase detectable similarity between homologous genes. In this paper, we introduce new tools to address this problem.

## Background

In 1984, Katsura and Hendrix [1] showed that when a specific gene of the phage λ was shortened, the resulting viruses’ tails were proportionally shorter. The corresponding *tape measure protein* has since been identified in a large number of phages and prophages. These proteins often have a variable number of tandem repeats with highly conserved tryptophan (W) and phenylalanine (F) amino acids at fixed positions that are used as anchors by small auxiliary proteins to stretch the tape and scaffold the actual tail construction (see, for example, [2]). The regular spacing between these anchors, or *period*, seems to be a key structural property of the tape measure protein and acts as a marking on the tape.

Phages are believed to be, by far, the most abundant form of life on the planet [3], a fact reflected by the large number of phage and prophage genomes currently available. This wealth of data allowed us to literally shop for tape measures that had specific properties in terms of length, period, composition and level of similarity.

**Figure 1:**
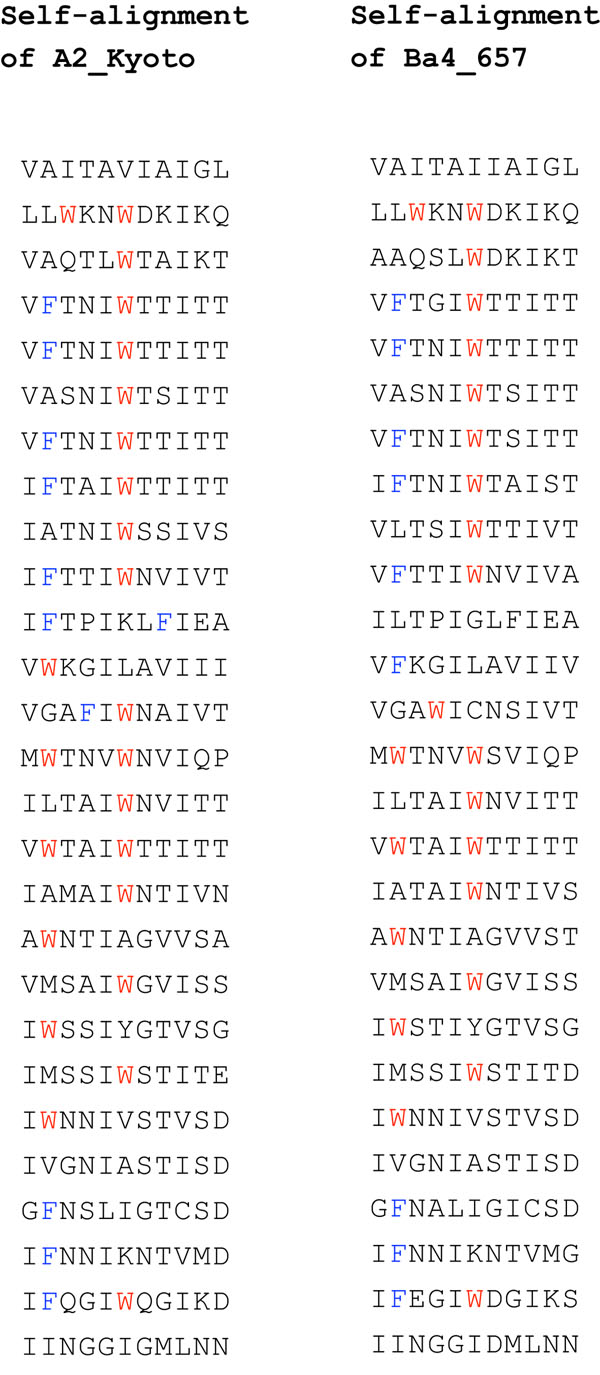
**Self and parallel alignments of tandem repeat sequences** Self and parallel alignments of two tape measure proteins. The prophage sequences are named after the strain of *Clostridium botulinum* in which they were found. Amino acids F and W are highlighted in the sequences.

Figure 1 gives a specific example of two repeat sections of tape measure proteins from prophages found in two strains of *Clostridium botulinum* genomes (accession numbers [YP_002803860:470..1294] and [YP_002862700:367..1191]). Each of the two sequences is self-aligned to show the 11 amino acids repeat, and the parallel gapless alignment of the two sequences shows the conservation across species, about 87% identity for the amino acid sequences, and 85 % for the underlying DNA sequences. The higher similarity of the orthologous segments (pairs of segments on the same line of the parallel alignment) compared to paralogous segments (pairs occurring in the same species) led us to conclude that these two sequences share the same duplication history. Additional File 1 contains a detailed discussion of this issue.

Reconstructing duplication histories has been an intensively studied combinatorial problem in the last ten years or so (see [4] and [5] for reviews), following an initial, more biology oriented work, by Walter Fitch in 1977 [6]. Recent advances on duplication history reconstruction extend the previous models by allowing more operations such as inversions [7] or segmental duplications [8]. All these approaches suppose a *fixed boundaries* model, meaning that duplication events may only occur at a fixed set of breakpoints, that does not apply very well to virus duplications (see Additional File 2). However, the basics of the theory of reconstructing unrestricted duplications was developed by Benson and Dong [9] in 1999, and constitutes the starting point of the present study. The idea of their heuristic is to evaluate the number of mutations for each putative duplication event, and choose to contract the segment with minimum, or near minimum, number of mutations.

We tested most available algorithms and heuristics - with or without the fixed boundaries hypothesis -using the amino acid sequences and the corresponding DNA sequences of the two viruses in Figure 1. Unfortunately, despite the striking similarity of the sequences, the two versions of the duplication history of their presumed common ancestor were always different. Since their divergence, each virus seems to have added embellishments to the original story, by the way of mutations, that eventually blur the common origin of their duplication history.

Here we develop a method that uses, in parallel, the information from two or more sequences to detect the most recent duplication event of their ancestor. It is based on an algorithm that computes the expected number of mutations that occurred before any speciation event.

## Results and discussion

### The units of the tape measures

**Figure 2:**
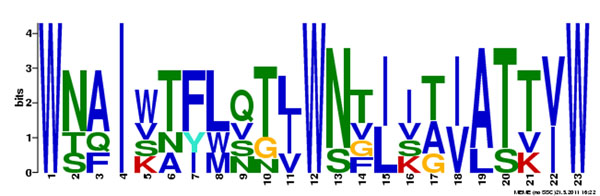
**Units on the tape** A motif, with 15 occurrences, generated by the Meme Motif Discovery software [12] using three tape measure protein sequences shows the period of 11 amino acids and tryptophan (W) as a “marker” on the tape.

The telltale of units in tape measure proteins is tandem repeat sequences, that can be detected with existing software [10,11]. However, since these tools are based on sequence similarity, that can be barely detectable in some cases, they must be complemented by alternative tools. Figure 2 shows a motif generated by the Meme Motif Discovery software [12] with three tape measure proteins of *Staphylococcus* phages SAP-26, 69 and D139 (accession numbers [YP_003857082], [YP_239580] and [ZP_0632492l]). This motif indicates a possible repeat unit of 11 amino acids, with the amino acid tryptophan (W) as a marker.

The structural analysis of Siponen et al [2] suggested that amino acid phenylalanine (F) could be an alternate marker, and that a pattern with mixed period (11-11-18) could also be present. This information was used to construct two search patterns - in ProSite format:

Pattern 1: [FW]-x(10)-[FW]-x(10)-[FW]-x(10)-[FW]-x(10)-[FW]-x(10)-[FW]-x(10)-[FW]

Pattern 2: [FW]-x(10)-[FW]-x(10)-[FW]-x(17)-[FW]-x(10)-[FW]-x(10)-[FW]-x(17)-[FW]

Using the BLAST package algorithm **seedtop** (ftp://ftp.ncbi.nlm.nih.gov/blast) we found that Pattern 1 has occurrences in 191 of the 5608 proteins records in Genbank that have an explicit reference to “tape measure phage protein”, and Pattern 2 has occurrences in 102 of the 5608 proteins (as of April 22, 2011). Of these, 16 sequences have occurrences of both patterns, yielding a total of 277 sequences that contain at least one occurrence of either pattern, or nearly 5% of the 5608 sequences. Note that these results poorly reflect the real number of tape measure proteins with such periods, since many proteins highly similar to known tape measure proteins are annotated with various descriptors that range from “minor tail protein” to “hypothetical protein”.

This was an encouraging first result that yielded examples of tandem repeats that are discussed in the next paragraphs. However, further investigations, both computational and biological, are needed to discover, if they exist, the repeated units of the remaining annotated tape measure proteins. Current automated tandem repeat finders rely on internal similarity to identify repeated units, and many tape measure proteins fail to show them. Biological evidence of conserved structures - such as the work described in [2] -are key observations that allow to construct alignments using these structures, but are based on protein crystallography experiments, thus not widely available.

### Reconstruction of the duplication history

**Figure 3:**
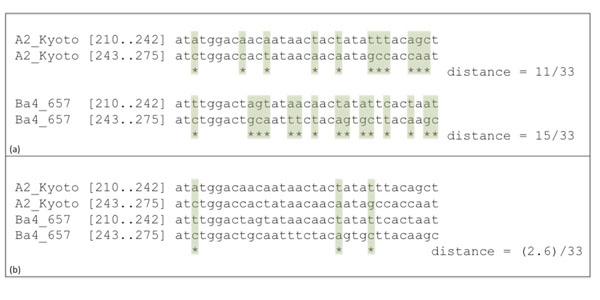
**Examples of distance computations** Part (a) shows the computation of normalized distance between two pairs of segments. The distance is the number of positions with different nucleotides divided by the length of the sequence. Part (b) shows the new normalized distance using information from the four segments. This distance is computed by evaluating the number of mutations that precede speciation, assuming that the speciation event followed the duplication event. (See Methods for the details of the computation).

We initially tried to reconstruct the duplication history of the two phage DNA sequences that code for the sequences in Figure 1 by applying the Benson & Dong algorithm [9] separately to each sequence. The algorithm computes a *normalized distance* between each tandem pair of segments, and chooses as the most plausible recent duplication a pair that minimizes the distance. The normalized distance is obtained by computing the number of mutations necessary to transform one segment into the other, normalized by dividing by the length of the segments. Minimizing this distance yields a most parsimonious duplication event with respect to the average number of mutations necessary to explain it. (For further details, see the Method section.) For example, comparing the two consecutive segments of length 33 starting at position *p* = 210 yield the normalized distances shown in Figure 3(a).

**Figure 4:**
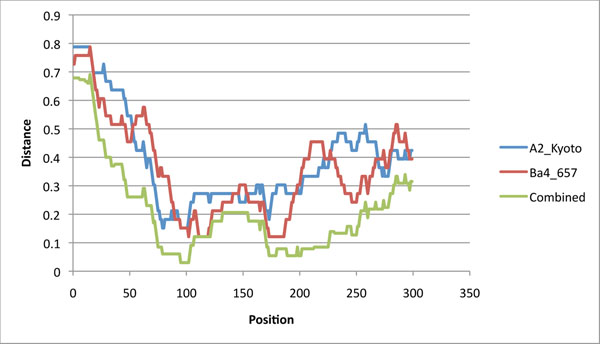
**Evaluating the position of the most recent duplication** Each of the three curves evaluates the cost of a duplication of length 33 at position *p* along the sequence of tape measure genes. The two top curves, obtained by independently computing the distances using the two orthologous sequences of Figure 1, do not even agree on positions where minimum cost occur. The bottom curve combines the information of the two sequences by discriminating between “recent” mutations, and mutations that occurred before the speciation event.

The Benson & Dong algorithm requires these distances to be evaluated for each possible position and each possible multiple of the period, here 33 base pairs. In this first experiment, both sequences predict that the most recent duplication is a segment of length 33 nucleotides, but disagree on where it should be. The two top curves of Figure 4 plot the distance versus position for the two phage sequences: the curves often widely disagree, including on where the minima are attained. The graph for phage A2_Kyoto reaches its minimum at each position in interval [95..101], and the graph for phage Ba4_657, at position 102, and in intervals [111..119] and [173..187].

Assuming that the two sequences are indeed orthologous, the origin of disagreements between the curves lies in mutations that occurred after speciation. Consequently, if the data of both sequences are to be used to reconstruct the duplication history, it is necessary to develop a scoring technique, detailed in the Methods section, that can discriminate between “recent” mutations and “ancient” mutations.

Contrary to the classic distance that counts the number of positions in which two sequences are different, the new distance is based on the simultaneous comparison of four sequences. An example of computation taken at position *p* = 210 is shown in Figure 3(b). In this example, only three columns have a positive score: the first and third columns contain the motifs *actc* and *tgtc* and get a score of 0.8, reflecting the expected number of mutations that preceded the speciation event; the second column contains the motif *tata* and gets a score of 1. The *combined* normalized distance is thus (2.6)/33.

This combined normalized distance applied to all possible positions yields the bottom curve of Figure 4. The new curve smooths out the differences between the first two curves, and narrows the search for the position of the most recent duplication: it reaches its minimum value at each position of interval [97..102]. This approach can be used recursively in order to reconstruct the recent duplication history of these sequences (data not shown) but going further might stretch too far the use of a heuristic on limited input. However, pinpointing the possible positions of the most recent duplication can be useful in establishing the phylogenetic relationships between tape measure proteins, as we show in the next section.

### Duplication or loss?

**Figure 5:**
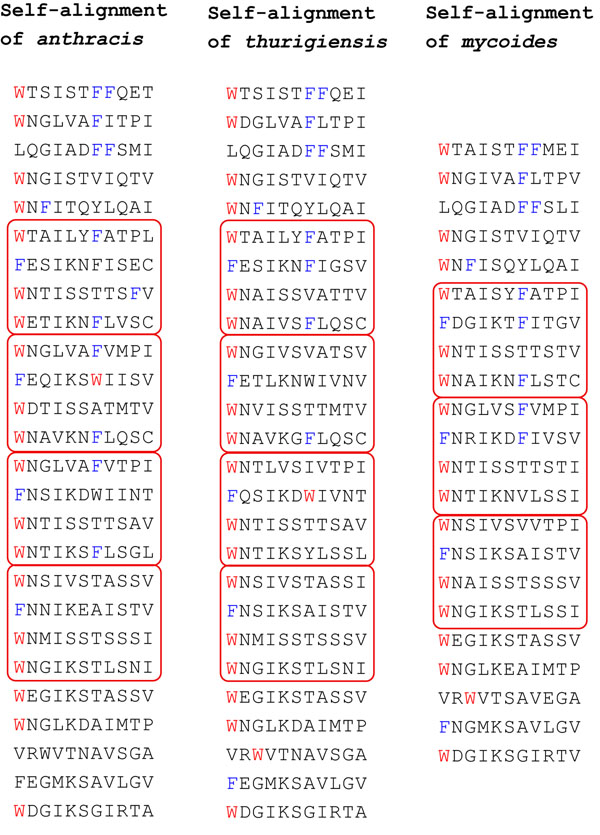
**Duplications or losses in tape measure proteins** Three tape measure proteins of different lengths: an apparent block of 44 amino acids is repeated four times in the first two sequences, and three times in the third one. Each sequence comes from of a prophage genome, and is named after the strain of *Bacillus* where it was found.

We now turn to a group of closely related phages that infect bacteria of the *Cereus* group. Figure 5 shows the self-alignments of three tape measure proteins from prophages labeled by the strain of *Bacillus* in which they were found (accession numbers *anthracis* [NP_846030: 337..622], *thurigiensis* [YP_003664881: 223..508] and *mycoides* [ZP_04158128: 746..987]). One of them, *mycoides*, is shorter than the others by 44 amino acids. The heuristic of the preceding section applied to *anthracis* and *thurigiensis* predicts that the most recent duplication of their common ancestor is 132 nucleotides long, or 44 amino acids. It is thus natural to conjecture that *mycoides* is a descendant of the *pre-duplicated* ancestor. However, there is always the possibility of a loss of a bloc of 44 amino acids in an ancestor of *mycoides.*

When two tandem repeat sequences are suspected to differ by one duplication or loss event, it is possible to estimate at which position this event occurred, regardless of the nature of the event (see Methods for the theoretical aspects). If the most recent event is a duplication event, its position can be determined by the techniques of the preceding section. In theory, there are two cases:

1. The two predictions agree. Then the most recent event is either a duplication, or a loss of a recently duplicated segment.

2. The two predictions disagree. Then the most recent event is a loss.

**Figure 6:**
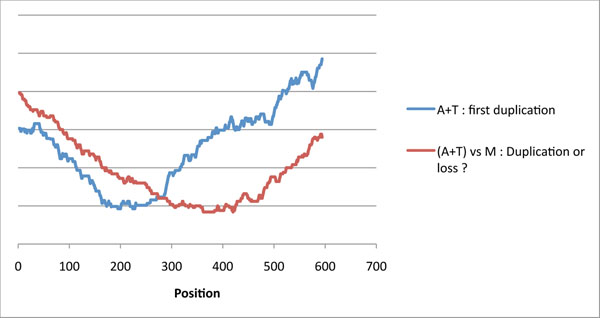
**Discriminating between duplications or losses** The blue curve (A+T recent duplication) evaluates the cost of the most recent duplication of the ancestor of *anthracis* and *thurigiensis* being at position *p.* The red curve evaluates the cost of a duplication/loss event at position *p* when comparing the consensus of *anthracis* and *thurigiensis* to *mycoides.* The intervals where these costs are minimal, or near minimal, are disjoint..

Figure 6 shows the two sets of predictions: the curve giving the cost of a duplication at position *p* for the combined sequences of *anthracis* and *thurigiensis;* and the curve indicating the cost of a duplication/loss event at position *p* when comparing *mycoides* to the consensus of *anthracis* and *thurigiensis.* The two curves reach their minimal, or near minimal, values in disjoint intervals, giving more weight to the hypothesis that the event was a loss rather than a duplication.

This result illustrates the difficulty of deciding between duplication and loss. Indeed, as simulations show (see the Methods section), the shape of the curve that determines the position of the most recent event is also an indication of its nature.

## Methods

### Heuristic for duplication reconstruction

The duplication reconstruction heuristic proposed in [9] compares every possible pair of consecutive segments of length *np* where *n* is an integer greater than 0, and *p* is the period of the repeat. Each comparison results in a score that is divided by *np*, and the duplication with the lowest score is a candidate for *contraction.* A contraction merges together the two consecutive segments by using the Fitch *procedure:* let *S* and *T* be the sets of nucleotides at position *j* in each segment, if *S* ∩ *T* ≠ ∅, then the new position *j* is filled by *S* ∩ *T*, otherwise it is filled by *S* ∪ *T.*

In the original paper of Benson and Dong, the sequences were scored by the number of unions performed in the comparison, which is proportional to the number of mutations that separates the two segments. In this paper, we want to apply the same heuristic, but with a different scoring technique that uses two or more DNA sequences whose common ancestor underwent the duplication events. To do this, we must be able to evaluate the number of mutations that occurred before the speciation event(s).

### Orthologous and paralogous nucleotides

The self-alignments of Figure 1 are gapless, and this property holds also for the alignment of the underlying DNA sequences. This allows us to apply the classical terminology of paralogs and orthologs to single nucleotide positions.

Suppose that a sequence of length *p* undergoes a series of duplications of lengths *np*, where *n* is an integer greater than 0. For example:

The length of the resulting sequence will also be a multiple of *p*, and any two nucleotides in the resulting sequence whose positions differ by a multiple of *p* were created by a duplication event, thus can be called *paralogs.* In our model, two tape measure proteins that have a good parallel alignment, such as the one in Figure 1, are presumed to share the duplication history of their common ancestor. Under this hypothesis, all duplications occurred *before* the speciation event, and nucleotides that are in the same respective position in each sequence can be called *orthologs.*

**Figure 7:**
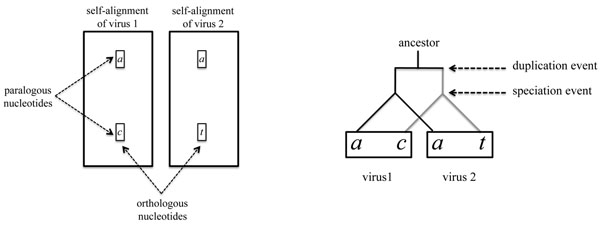
**Orthologs and paralogs** Paralogous nucleotides are in the same column of a single alignment, orthologous nucleotides are in the same position of a parallel alignment. If two pairs of orthologs are in the two same columns of a parallel alignment, their relations can be captured by a Fitch diagram.

Figure 7 shows the orthology and paralogy relations among four nucleotides, and the corresponding Fitch diagram depicting the duplication and the speciation events. Given such a diagram, whose leaves are labeled by a *motif x*_1_, *y*_1_, *x*_2_, *y*_2_ of 4 nucleotides, a first problem is the following:

**Problem 1** Suppose that a duplication event created paralogous nucleotides *x* and *y*, and that a subsequent speciation event created orthologous viruses 1 and 2, yielding the two pairs of orthologs *x*_1_ and *x*_2_, and *y*_1_ and *y*_2_, what is the expected number of mutations that occurred *before* the speciation event?

### F-trees and the Fitch algorithm

**Figure 8:**
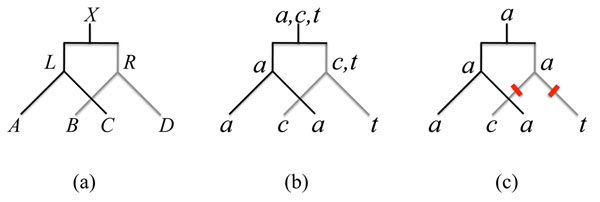
**F-trees, Fitch sets and labelings** (a) An F-tree. (b) An example of Fitch sets with *N* = 2 unions. (c) A labeling of the tree (b) with *N* = 2 mutations. Note that the label of the right node in tree (b) does not belong to the corresponding Fitch set in tree (a).

To discuss the properties of the model, we need the following definition and notations, illustrated in Figure 8.

**Definition 1*** An* F-tree *is a duplication-speciation tree with 4 ordered leaves labeled by sets A*, *B*, *C*, *D that are subsets of the set of nucleotides* {*a*, *c*, *g*, *t*}*.*

• *The* left *node is the parent of the leaves labeled by sets A and C. It is labeled by L* = *A* ∩ *C*, *if this set is non-empty*, *otherwise by L* = *A* ∪ *C.*

• *The* right *node is the parent of the leaves labeled by sets B and D. It is labeled by R* = *B* ∩ *D*, *if this set is non-empty*, *otherwise by R* = *B* ∪ *D.*

• *The* ancestor *node is labeled by X* = *L* ∩ *R*, *if this set is non-empty*, *otherwise by X* = *L* ∪ *R.*

The number *N* of mutations of an F-tree is the number of set unions necessary to construct the sets *L*, *R* and *X.* A *labeling* of an F-tree is a labeling of its leaves and nodes by nucleotides, such that each leaf is labeled by a nucleotide that belongs to its set label, and such that the number of mutations - that is, edges with different labels at their extremities - is equal to *N.* Note that it is not mandatory that nucleotides that label internal nodes belong to the corresponding set label, *L*, *R* or *X.*

The procedure outlined in Definition 1 was originally proposed be W. Fitch [13] as a way to compute the minimum number of mutations for a given tree, it was later proven correct by D. Sankoff [14]. The sets computed for a parent node by this rule are called *Fitch sets.*

A mutation occurs *before* the speciation event in a given labeling if it occurs between the root and one of its children, otherwise it occurs *after* the speciation event. If an F-tree has several labelings, we denote by *N_b_* the average number of mutations that occurs before speciation among all possible labelings, and by *N_a_*, the average number of mutations that occurs after speciation. Clearly, *N_b_* + *N_a_* = *N.*

We first compute, as an example, the values of *N* and *N_b_* in the case all sets *A*, *B*, *C* and *D* are singletons. The general proof is presented in the next section. There are 4^4^ = 256 different motifs of 4 nucleotides. With respect to our problem, they can be partitioned into 7 classes with the following representatives: *aaaa*, *aaat*, *atta*, *caat*, *tata*, *acat*, and *actg.* Of these, the first four cases yield *N_b_* = 0.

**Figure 9:**
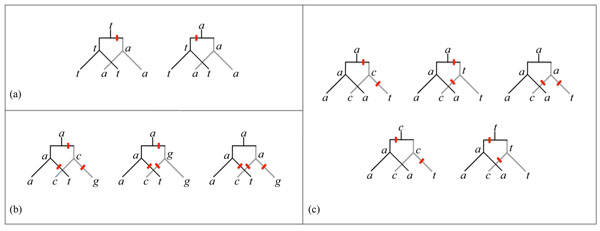
**Possible labelings of selected motifs** (a) The two possible labelings with one mutation of the *tata*-motif imply that the mutation occurred before the speciation event. (b) Two out of three labelings of the *actg-*motif imply a mutation event before the speciation event. Only the labelings with nucleotide *a* as an ancestor are shown, the 3 other cases are similar. (c) Four of the five possible labelings of the *acat*-motif tree contain a mutation event before the speciation event.

The *tata*-motif is the simplest of the remaining cases, since only one mutation is required to generate it. This motif can be described as two pairs of equal orthologous nucleotides. Figure 9(a) shows the two possible labelings, and the single mutation can only be assigned before the speciation event.

The *actg*-motif, on the other hand, requires a minimum of three mutations. Figure 9(b) shows 3 of the 12 possible labelings and, on average, 2/3 mutations occur before the speciation event, and 7/3 after. Note that the third labeling is not obtainable by the Fitch traceback algorithm since the label of the right child of the root is not contained in the union of the labels of its children.

The *acat-*motif is the most complex and is shown in Figure 9(c). It has one pair of equal orthologous nucleotides, and requires a minimum of two mutations. Three labelings have nucleotide *a* as an ancestor, one has nucleotide *c* and one has nucleotide *t.* On average, 4/5 mutations occur before the speciation event, and 6/5 after.

In the next sections, we will show how these observations can be generalized to trees labeled by sets.

### Computing the average number of mutations preceding speciation

When the leaves of an F-tree are labeled by sets containing more than one element, the possible labelings can include more than one motif. For example, if:

*A* = {*a*, *t*}, *B* = {*a*, *t*}, *C* = {*t*}, *D* = {*a*}

then the Fitch procedure yields *N* = 1 mutations. Four possible labelings achieve this minimum: two with *tata* labeling the leaves, one with *aata*, and one with *ttta.* The motif *atta* is excluded since it requires *N* = 2 mutations.

In order to solve the general case, given the sets *A*, *B*, *C* and *D*, consider the following parameters:

The next three lemmas give the average number of mutations that occur before speciation in an F-tree with leaves labeled by the sets *A*, *B*, *C* and *D*, with *N* > 0 minimum number of mutations:

**Lemma 1 ***When N* = 1, *the average number of mutations that occur before speciation is given by:*

*Proof.* There is only one mutation when exactly one of the sets *A* ∩ *C*, *B* ∩ *D* or *L* ∩ *R* is empty. If *L* ∩ *R* is not empty, then either *A* ∩ *C* = ∅ or *B* ∩ *D* = ∅. If *A* ∩ *C is* empty, then the single mutation occurs in the left subtree, there is at least one motif with three equal nucleotides implying *n_aaat_* > 0, *n_tata_* = 0, and *N_b_* = 0, thus the result holds. The case *B* ∩ *D* is similar.

If *L* ∩ *R* is empty, then any motif with two different nucleotides may be present, but only motifs with orthologous equal nucleotides (the *tata*-motif), or motifs with three equal nucleotides, yield *N* = 1. The *tata*-motif has two different labelings, as seen in Figure 9(a), both of which assign the mutation before the speciation event. The *aaat*-motif has only one labeling, and the mutation occur after the speciation event. Thus *N_b_* = 2*n_tata_/*(2*n_tata_* + *n_aaat_*)*.*

**Lemma 2*** When N* = 2, *the average number of mutations that occur before speciation is given by:*

*Proof.* We first consider the case *L* ∩ *R* ≠ ∅. Both *A* ∩ *C* = ∅ and *B* ∩ *D* = ∅, implying *n_acat_* = 0. Since [(*A* ∪ *C*) ∩ (B ∪ *D*)] ≠ ∅, then at least one of the sets *A* ∩ *B*, *A* ∩ *D*, *C* ∩ *B* or *C* ∩ *D* is not empty. In this case, *n_caat_* may be 0 when both |*A* ∩ *B*| and |*C* ∩ *D*| are non zero, or both |*A* ∩ *D*| and |*C* ∩ *B*| are non zero, but in these cases, *n_atta_* > 0, thus we have (2*n*_atta_ + *n_caat_*) > 0. The *atta*-motif has two possible labelings, both of which assign the two mutations after speciation, and the *caat*-motif has only one labeling, also with the two mutations after speciation, thus *N_b_* = 0 and the formula holds.

When *L* ∩ *R* = ∅, then one of *A* ∩ *C* or *B* ∩ *D* is not empty and *n_acat_* > 0. As seen on Figure 9(c), there are five possible labelings of *acat-*motifs, four of which have a mutation preceding speciation. However, *atta*-motifs and *caat*-motifs may also be present, for example with:

*A* = {*a*, *t*}, *B* = {*c*, *g*}, *C* = {*a*, *g*}, *D* = {*t*}.

Thus, *N_b_* = 4*n_acat_*/(5*n_acat_* + 2*n_atta_* + *n_caat_*).

**Lemma 3 ***When N* = 3, *the average number of mutations that occur before speciation is given by N_b_* = 2/3. *Proof.* In order to have *N* = 3, all three sets *A* ∩ *C*, *B* ∩ *D* and *L* ∩ *R* = (*A* ∪ *C*) ∩ (*B* ∪ *D*) must be empty, thus the four sets are singletons, and, by the case study of Figure 9(b), *N_b_* = 2/3.

### Detecting duplication and loss events

In this section, we discuss the problem of detecting a duplication or loss event when comparing two tandem repeat sequences. We first discuss this problem in the fixed boundary context. Formally, we are given two sequences:

*b* = *b*_1_…*b_j_*_–1_*b_j_*_+1_…*b_n_*

*c* = *c*_1_…*c_j_*_–1_*c_j_c_j_*_+1_…*c_n_*

each of them composed of segments of the same length, and both sharing a common ancestor *a* that contained all segments, except possibly the segment at position *j.* The relation between the sequences is either a duplication creating segment *c_j_* in the lineage of sequence *c*, or a loss of segment *b_j_* in the lineage of sequence *b.* The Hamming distance between two segments is denoted by *H*(*s*, *t*) and measures the number of position with different nucleotides in segments *s* and *t.* Under these hypothesis, the problem is the following:

**Problem 2** Given sequences *b* and *c*, what is the position *j* of the duplication or loss event that minimizes the distance between the sequences?

Define *c*|_*i*_ = *c*_1_*…c_i_*_−1_*c_i_*_+1_…*c_n_*, as the sequence *c* with segment at position *i* removed. Then we have:

**Proposition 1** If *H*(*b_i_*, *c_i_*) ≤ *H*(*b_i_*, *c_ℓ_*), for ℓ ≠ *i*, then the function *H*(*b*, *c*|*_i_*) attains a minimum when *i* = *j*.

*Proof.* We have . If *i* <*j* then

thus, since *H*(*b_k_*, *c_k_*) ≤ *H*(*b_k_*, *c_k_*_+1_), we have:

The same reasoning holds when *i* >*j.*

The hypothesis that *H*(*b_i_*, *c_i_*) ≤ *H*(*b_i_*, *c_ℓ_*) reflects the fact that the duplication event(s) that created the segments at position *i* and ℓ preceded the speciation event that created sequences *b* and *c*. In real data, the hypothesis might not hold for all values of *i* and ℓ, but it should hold on average.

Without the assumption of repeats with fixed boundaries, it is still possible to use Proposition 1 to obtain an estimate of the position of a duplication or loss event by testing all possible sets of boundaries. This is equivalent to computing, for each position *i* of the nucleotide sequence *c*, *H*(*b*, *c*|_[_*_i_*_,_*_i_*_+_*_d_*_)_), where *d* is the difference in length of the two sequences, and *c*|_[_*_i_*_,_*_i_*_+_*_d_*_)_ is the sequence *c* with all nucleotides between positions *i* and *i* + *d* – 1 removed.

**Figure 10:**
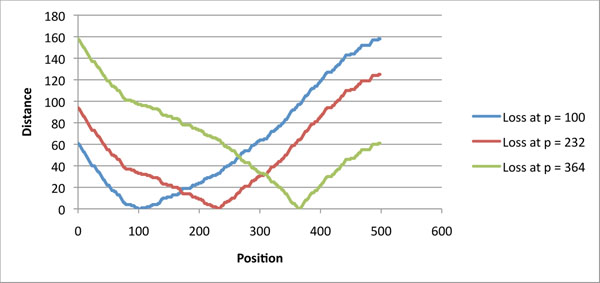
**Simulation of loss events** Three different loss events were simulated in the prophage tape measure gene of A2_Kyoto. The graphs of the function *H*(*c*|_[_*_p_*_,_*_p_*_+33)_, *c*|_[_*_i_*_,_*_i_*_+33)_) exhibit a clear minimum around the corresponding values of *p*, at position 100, 232 and 364. Position 100 corresponds to a loss at the position of the most recent duplication. When the loss event is far from the position of the most recent duplication, the curve is markedly sharper near the minimum.

We also simulated loss events in prophage A2_Kyoto of Figure 1, whose most recent duplication, according to the graph of Figure 4, occurs around position *p* = 100 and is of length 33. Figure 10 shows the graph of function *H*(*c*|_[_*_p_*_,_*_p_*_+33)_, *c*|_[_*_i_*_,_*_i_*_+33)_) for three different loss events, one at *p* = 100, one at *p* = 232 and one at *p* = 364. Each curve exhibits a clear minimum around the position of the simulated loss event, but the shape of the curve differs depending on the distance between the position of the loss event and the position of the most recent duplication.

## Conclusions

In this paper, we developed a variety of tools to study the evolution of tape measure proteins. We relied on existing software to identify repeated units and markers, and we have already identified hundreds of sequences that have a clear repetitive structure. However many tape measure proteins do not have readily identifiable repeat sequences, or markers, and new methods must be developed to classify them.

In order to study the duplication histories of this first set of sequences, we developed new theoretical tools that could use in parallel the information provided by slightly divergent sequences. For the time being, these analysis are restricted to pairs of sequences for two main reasons: (1) the algorithm assumes an established rooted phylogeny of the studied sequences, and, given the high rate of recombinations between phages [15,16], this is not a trivial task; (2) the computational complexity of extending the algorithm to more than two species is unknown, but suspected to be hard.

## Competing interest

The authors declare that they have no competing interests.

## Supplementary Material

Additional file 1 **Uncovering shared duplication history** Techniques for detecting shared duplication history between tandem repeat sequences. Click here for file

Additional file 2 **Models for boundaries in tandem repats** The fixed boundaries model and the unrestricted boundaries model for tandem repeat sequences Click here for file
