# Supplementary material for: The evolution of the tape measure protein: units, duplications and losses
Source: BMC Bioinformatics. 2011 Oct 5;12(Suppl 9):S10. doi: 10.1186/1471-2105-12-S9-S10 (PMC3271669; doi:10.1186/1471-2105-12-S9-S10)
Supplement: Additional file 1 — Uncovering shared duplication history Techniques for detecting shared duplication history between tandem repeat sequences. [file 1471-2105-12-S9-S10-S1.pdf]

Additional File 1

Uncovering shared duplication history

Two tandem repeat sequences of the same unit may share their whole duplication history, or only part of it, as further duplication events may occur independently in each lineage after divergence. Deciding between the two hypotheses rely on various characteristics of the sequences, and evidence for a common duplication history can be accumulated by identifying the following conditions:

(1) The two sequences are highly similar, have the same number of repeats, and are flanked by highly similar regions.

(2) Both sequences, when queried independently, trace their most recent duplication event to be nearly the same length ℓ.

(3) Both sequences, when queried independently, trace their most recent duplication event at nearly the same positions.

(4) When both sequences are cut into segments of length ℓ, orthologuous segments across species are much more similar to the neighboring paralogous segments within each species. (This is what we call *parallel alignments* in the main text.)

These four tests were our road map for assessing the hypothesis that the sequences shown in Figure 1 shared their whole duplication history.

Condition (1) was easily resolved, since the sequences have 87% identity for their amino acid sequences, and 85% for the underlying DNA sequences. Both sequences have the same length and the alignments have no gaps.

When queried by the Benson and Dong algorithm [9], both sequences predicted that their most recent duplication had length 33 nucleotides, or 11 amino acids. However, they disagreed on the position(s) of this most recent duplication. The extent of these disagreements became the focus of our investigation.

In order to test Condition(4), we cut each sequence coding for the proteins of Figure 1 of the main text into 25 segments of 33 nucleotides, and using two flanking sequences of 33 nucleotides, for a total of 27 segments for each species, named K1 to K27 for the A2_Kyoto prophage gene, and B1 to B27 for the Ba4_657 prophage gene.

The crucial test for shared duplication history is that orthologous segments – segments in the same positions in the two sequences – should be more similar than paralogous segments – segments in the same sequence but in different positions. We constructed a neighbor-joining tree of the 54 segments, using Hamming distance and the tools available at the site `http://www.phylogeny.fr/` [Dereeper, 2008].

The tree shows that the nearest neighbors are the corresponding ortholog pairs in 22 out of 27 pairs. The exceptions are the pairs (K4, B4), (K5, B5), (K7, B7), (K8, B8), (K9, B9), shown in black in Supplementary Figure A.

Interestingly, this set of pairs contain both the most similar sequences, and the most dissimilar. Supplementary Figure B (a) shows the distances between orthologous and paralogous pairs from segment 4 to segment 7. These segments are very similar, and included, for both prophages, the predicted positions of the most recent duplication. The fact that, for each pair, the similarity is higher or equal for orthologs than for paralogs is a further indication that the sequences share their duplication history in this region rather than having undergone two independent duplications.

The case of pairs (K8, B8) and (B9, K9) is shown in Supplementary Figure B (b). In this case, the distances between orthologs is larger than some of the distances between paralogs, indicating a possible case of parallel independent duplications. However, since all the distances are quite large, under the parsimony hypothesis, these events should be ancient. Thus we again conclude that events leading to these segments preceded speciation.

[Dereeper, 2008] Dereeper A., Guignon V., Blanc G., Audic S., Buffet S., Chevenet F., Dufayard J.-F., Guindon S., Lefort V., Lescot M., Claverie J.-M., Gascuel O. *Phylogeny.fr: robust phylogenetic analysis for the non-specialist Nucleic Acids Research.* 2008 Jul 1; 36 (Web Server Issue):W465-9. Epub 2008 Apr 19.

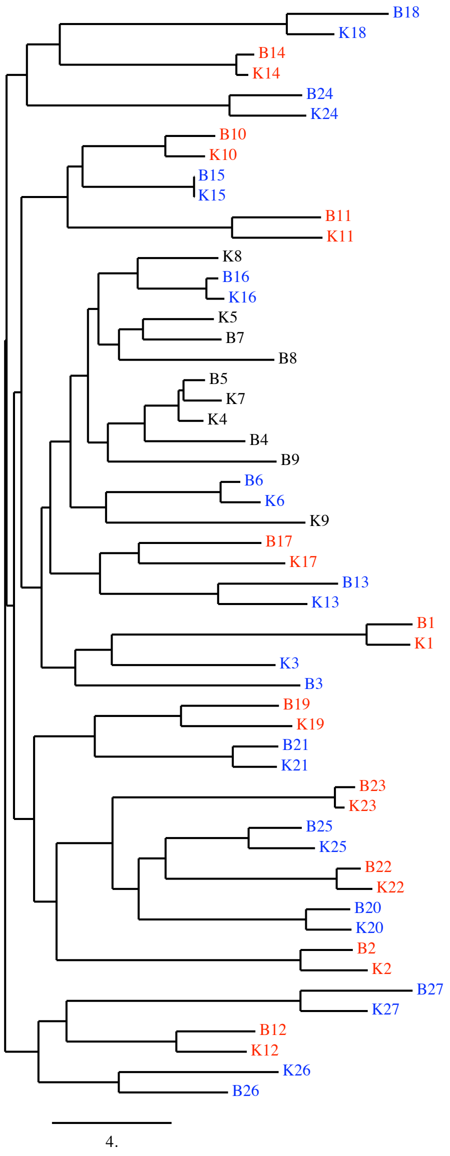

Fig. A. Phylogenetic tree of the 54 nucleotide segments coding for the 54 amino acid segments of Figure 1 of the main text. Each segment of the A2_Kyoto prophage is tagged by Ki where i is its line number, and the corresponding orthologous segment in Ba4_657 prophage is tagged by Bi. Out of 27 orthologous pairs, 22 are nearest neighbors, and are colored red or blue. The remaining five pairs are in black.

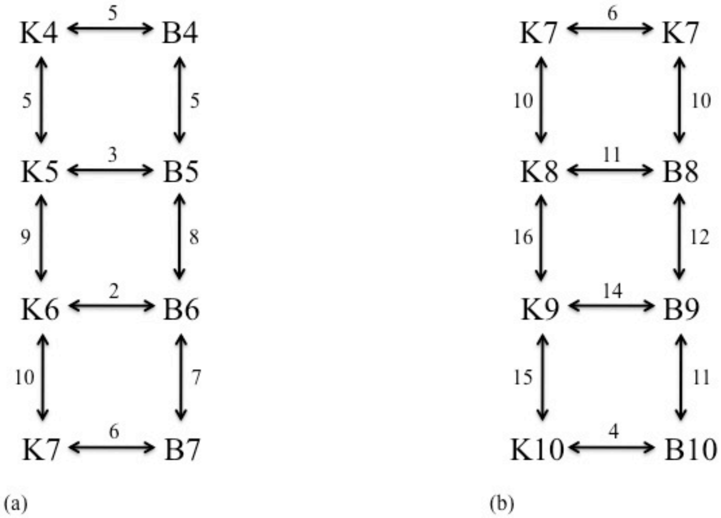

Fig. B. (a) Distances between orthologous and paralogous pairs from segment 4 to segment 7. Distances between orthologs are all smaller or equal to distances between paralogs. (b) Distances between orthologous and paralogous pairs from segment 7 to segment 10. The distance between segments K9 and B9 is larger than the distance between B8 and B9, and the distance between B9 and B10. This could indicate parallel independent duplications, but the large distances – compared to the distances between other pairs of orthologs – also indicate that these sequences may have diverged before speciation.
