# Supplementary material for: The evolution of the tape measure protein: units, duplications and losses
Source: BMC Bioinformatics. 2011 Oct 5;12(Suppl 9):S10. doi: 10.1186/1471-2105-12-S9-S10 (PMC3271669; doi:10.1186/1471-2105-12-S9-S10)
Supplement: Additional file 2 — Models for boundaries in tandem repats The fixed boundaries model and the unrestricted boundaries model for tandem repeat sequences [file 1471-2105-12-S9-S10-S2.pdf]

Additional File 2

Models for boundaries in tandem repats

The most general model of tandem repeat formation is the *unrestricted boundaries model* that does not put constraints on the locations of the breakpoints of a duplication/loss event.

For example, let *abcd* be a repeated unit, and consider the following three duplication events:

$$\begin{aligned} \underline{abcd} &\to ab\underline{cdab}cd & (1) \\ &\to a\underline{bcdabcda}bcd & (2) \\ &\to abcdabcdabcdabcdabcd & (3) \end{aligned}$$

The breakpoints of the first duplication are at the extremities of the repeated unit, ie. before symbol a and after symbol d; the breakpoints of the second are between symbols b and c; and the breakpoints of the third are between symbols a and b.

On the other hand, the *fixed boundaries model* introduce the restriction that breakpoints are always located at the same relative position in the repeated unit.

For example, consider the following three duplication events:

$$\begin{aligned} \underline{abcd} &\to abcd\underline{abcd} & (4) \\ &\to \underline{abcdabcd}abcd & (5) \\ &\to abcdabcdabcdabcdabcd & (6) \end{aligned}$$

All breakpoints are located before symbol a or after symbol d, or between these two symbols. The repeated unit is never broken anywhere else, but the resulting sequence is exactly the same as in line (3).

When the fixed boundaries model holds, then the duplication history can be reconstructed using trees that encode the nature and order of the duplication events, and many tools based on phylogeny of the repeated units have been developed to reconstruct the duplication history (see [4] for a review of all existing techniques).

With the unrestricted model, the duplication history is encoded by a more complex structure than a tree, and phylogeny tools are not directly applicable. To our knowledge, the only approach that applies to this model is the Benson and Dong heuristics [9].

On the relevance of the fixed boundaries model with respect to real data, Rivals [5] notes that: “Most researchers envisaged the history problem [...] with this restriction, since they consider the case of tandemly repeated genes where it seems to apply.”, with the further remark that: “From the biological point of view, many tandem repeats do have not an integer, but rather a truly rational number of copies, showing that boundaries of amplifications vary.”

Thus, for the fixed boundaries model to apply, biological evidence must be provided and may consist in:

(1) An integral number of units in the tandem repeat sequence.

(2) When the repeated units are genes, large intergenic regions separating the units may indicate that the breakpoints lie in these regions.

(3) Clear identification of breakpoint regions, that can be pinpointed by non-congruent phylogenies ([Grassly, 1997], [McGuire, 1997]).

In the case of the tape measure protein discussed in this paper, no such evidence has yet been found. The number of units cannot be determined exactly since the sequence containing the repeated units is embedded in a larger protein (see [2]). Furthermore, since the repeated units are small, with no gaps or flanking sequences between the units, breakpoint identification techniques are hard to apply.

[Grassly, 1997] Grassly, N. C. and Holmes, E. C., *A likelihood method for the detection of selection and recombination using nucleotide sequences*, Mol. Biol. Evol., 1997, 14, 239–247.
[McGuire, 1997] McGuire, G. and Wright, F. and Prentice, M. J., *A graphical method for detecting recombination in phylogenetic data sets*, Mol. Biol. Evol., 1997, 14, 1125–1131.
